# Morphological parameters of myopic choroidal neovascularization as predictive factors of anti-VEGF treatment response

**DOI:** 10.1038/s41598-022-14287-7

**Published:** 2022-06-21

**Authors:** Niccolò Castellino, Maurizio Battaglia Parodi, Andrea Russo, Mario Damiano Toro, Matteo Fallico, Vincenza Bonfiglio, Agatino Davide Maugeri, Teresio Avitabile, Antonio Longo

**Affiliations:** 1 Department of Ophthalmology, University of Catania, Via Santa Sofia 78, 95178 Catania, Italy; 2 Department of Ophthalmology, Ospedale San Raffaele, University Vita-Salute, Milan, Italy; 3 Department of General Ophthalmology, Medical University of Lublin, Lublin, Poland; 4 Department of Experimental Biomedicine and Clinical Neuroscience, University of Palermo, Palermo, Italy

**Keywords:** Predictive markers, Retina

## Abstract

The objective of this prospective study was to investigate the morphological changes of myopic choroidal neovascularization (mCNV) after treatment with anti-vascular endothelial growth factor and to identify potential features predictive of the final BCVA. OCT and OCTA features were evaluated at baseline and at 1, 6 and 12 months. Parameters investigated were the maturity pattern, presence of mCNV OCT activity signs, subretinal fibrosis and mCNV area. Forty patients (41 eyes) were included in the study. At the final visit, after a mean of 3.1 ± 1.4 injections, BCVA had improved significantly (p = 0.009) and subretinal hyperreflective exudation, subretinal fluid and intraretinal cysts nearly disappeared at 12 months. At baseline, 20 eyes had an immature CNV that were smaller, required less injections (2.5 ± 1.2 vs 3.8 ± 1.4, p = 0.002), they completely regressed in seven eyes and achieved a better BCVA (0.14 ± 0.15 vs 0.40 ± 0.26 logMAR, p < 0.001) when compared to mature CNV. Subretinal fibrosis developed in 19 eyes (46.3%) with lower final BCVA than eyes without fibrosis (0.19 ± 0.24 vs 0.38 ± 0.22 logMAR, p = 0.012). Baseline immature pattern (p = 0.005) and baseline BCVA (p < 0.001) were predictive of final BCVA. Multimodal imaging is useful to define mCNV changes during treatment. OCTA provides prognostic information which cannot achieved by other imaging techniques.

## Introduction

Pathologic myopia (PM) is characterized by a progressive eye elongation associated with degenerative chorio-retinal changes. The prevalence of myopia is increasing in Europe, North America and Asia, thus determining an increment of complications related to PM^1,2^.

Myopic choroidal neovascularization (mCNV) is a fearsome complication of PM, occurring in 5–11% of cases^3^, and representing a main cause of visual impairment in working people under the age of 50^4^.

The clinical presentation of mCNV on fundus biomicroscopy has characteristic features, including a slight and greyish retina elevation with limited exudation, central location, and restricted size. mCNV is a type 2 subform, typically growing above the retinal pigment epithelium and extending into the subretinal space^5,6^. The subretinal location allows better visualization than in type 1 subform, although both identification and activity assessment may prove deceptive in certain cases. The management of patients affected by active mCNV has been revolutionized by the advent of anti-vascular endothelial growth factor (anti-VEGF) injections. Randomized controlled trials have shown the efficacy and safety of anti-VEGF treatment with a limited number of injections. Fluorescein angiography (FA) represents the gold-standard to identify signs of mCNV activity and represents the yardstick to measure the accuracy of emerging imaging techniques in detecting CNV. Optical coherence tomography (OCT) plays an essential role in the diagnosis and follow-up of mCNV patients in a non-invasive fashion. Several OCT morphologic biomarkers of disease activity have been identified to simplify the management of mCNV patients.

Optical coherence tomography angiography (OCTA) is an imaging technique which directly visualizes the neovascular network. Several patterns of mCNV have been described based on their activity status, shape, maturation and fibrotic evolution^7–10^. Nevertheless, scant information is available regarding the correlation between OCTA characteristics and long-term anatomical and functional outcomes in treated mCNV.

The aim of this study is to report the morphological and functional changes of mCNV treated with anti-VEGF and to identify predictive factors by multimodal imaging over a one-year follow-up.

## Results

Overall, 41 eyes of 40 patients were included in the study (1 patient treated in both eyes); all patients concluded the study up to the 12-month examination (M12).

The 40 patients enrolled had a mean age of 57 ± 16.3 years (range 25–77), with 27 females. Complete baseline demographic and clinical characteristics of the study population are listed in Table 1.

**Table 1:** Baseline demographic and clinical characteristics of study population.

Patients’ characteristics (number of eyes = 41)	
Age, (years), mean ± SD	57 ± 16
Gender, n (male:female)	13:27
Eye Laterality, n (right:left)	19:22
CNV duration (days), mean ± SD	19.6 ± 6.8
Axial length (mm), mean ± SD	28.78 ± 1.31
Central foveal thickness (μ), mean ± SD	272 ± 42
Number of injections, mean ± SD	3.15 ± 1.46
Baseline BCVA, (logMAR), mean ± SD	0.48 ± 0.34
FA pattern, n (profuse:minimal)	23:18
CNV maturation pattern, n (immature:mature)	20:21
Subretinal haemorrhages, n (presence:absence)	5:36

*SD* standard deviation, *mm* millimeter, *BCVA* best-corrected visual acuity, *μ* micron, *CNV* Choroidal neovascularization, *logMAR* logarithm of the minimal angle of resolution.

At baseline, best-corrected visual acuity (BCVA) was 0.48 ± 0.34 logMAR; mean axial length was 28.78 ± 1.31 mm. Subretinal hyperreflective exudation (SHE) with fuzzy borders, ellipsoid zone disruption (EZ disruption), subretinal fluid (SRF) and intraretinal cysts (IRC) were seen respectively in 95%, 90.5%, 41% and 27% of the eyes.

Immature CNV was observed in 20 eyes and mature CNV in 21 eyes with a high agreement obtained between observers (Cohen’k = 0.903, p < 0.001). Immature mCNV size resulted in significantly smaller (0.18 ± 0.19 mm^2^ vs 1.01 ± 0.53 mm^2^, p < 0.001) and had a lower mean duration (16.1 ± 3.8 days vs 23 ± 7.4 days, p < 0.001) than mature mCNV. Mean Central Foveal Thickness (CFT) in immature and mature mCNV were 257 ± 35 μ and 288 ± 44 μ, respectively. No significant difference was found in axial length in eyes with mature and immature CNV (respectively 29.09 ± 1.43 mm and 28.46 ± 1.11 mm, p = 0.122).

All patients received their first ranibizumab injection, and all concluded the study, receiving the 12-month examination. The overall mean number of injections was 3.1 ± 1.4 (range 1–6).

BCVA significantly improved over the follow-up (p = 0.003, ANOVA test), in particular starting from 6-month examination (M6). Final BCVA was 0.28 ± 0.25 logMAR with a significant correlation between baseline BCVA and final BCVA (12 month) (Pearson, r = 0.863; p < 0.001).

Mean values of all parameters at different time-points are reported in Table 2.

**Table 2:** Results of repeated variables over the follow-up study period eyes with mCNV (n = 41).

Variable	baseline	1 Month	6 Months	12 Months	p value
BCVA (logMAR)	0.48 ± 0.34	0.34 ± 0.29	0.26 ± 0.24^a^	0.28 ± 0.25^b^	**0.003**^**A**^
Ellipsoid zone disruption (n)	37 (90.5%)	20 (61.9%) ^c^	3 (4.8%)^d^	6 (9.5%)^d^	** < 0.001**^**Q**^
Subretinal hyperreflective exudation (n)	39 (95.1%)	19 (46.1%) ^d^	3 (7.3%)^d^	1 (2.4%)^d^	** < 0.001**^**Q**^
Intraretinal cysts (n)	11 (26.8%)	10 (24.4%)	4 (9.8%)^e^	2 (4.9%)^f^	**0.002**^**Q**^
Subretinal fluid (n)	17 (41.5%)	3 (7.3%) ^g^	1 (2.4%)^d^	2 (4.9%)^d^	** < 0.001**^**Q**^
CNV area (mm^2^)	0.61 ± 0.58	0.54 ± 0.55	0.50 ± 0.47	0.49 ± 0.48	0.736^A^
Subretinal fibrosis (n)	0 (0%)	0 (0%)	10 (24.4%)^h^	19 (46.3%)^d^	**< 0.001**^**Q**^

Significant values are in bold.

^A^ANOVA test; ^Q^Cochran’s Q test; ^a^0.004 (Tukey HSD vs baseline); ^b^0.009 (Tukey HSD vs baseline); ^c^0.001(McNemar vs baseline); ^d^< 0.001(McNemar vs baseline); ^e^0.039 (McNemar vs baseline); ^f^0.012 (McNemar vs baseline); ^g^0.001 (McNemar vs baseline); ^h^0.002 (McNemar vs baseline).

*BCVA* Best-Corrected Visual Acuity, *CNV* Choroidal neovascularization, *logMAR* logarithm of the minimal angle of resolution, *mm* millimeter.

At the end of the follow-up (12 months), 21 mCNVs were classified as mature, 13 as immature and in 7 cases a complete mCNV regression was observed. Two baseline immature mCNVs (9.5%) developed into mature mCNVs. Conversely, 3 baseline mature mCNVs (15%) regressed to the immature form; 7 out of the 21 baseline immature CNVs (33.3%) underwent complete regression.

Patients affected by immature CNV received less injections than patients with mature CNV on average (2.5 ± 1.2 vs 3.8 ± 1.4, p = 0.002), and achieved a better final BCVA (0.14 ± 0.15 logMAR vs 0.40 ± 0.26 logMAR, p < 0.001). In both the groups, CFT decreased significantly compared to baseline (p < 0.001); mean final CFT in immature mCNV was 233 ± 26 μ and 254 ± 33 μ in mature CNV. In mature and immature groups, subretinal fibrosis (SF) at M6 was detected respectively in 9 eyes and 1 eye (p = 0.009), and M12 in 17 eyes and 2 eyes (p < 0.001).

At the final visit, subretinal hyperreflective exudation with fuzzy borders was detected in a single eye (2.4%), whereas subretinal fluid and intraretinal cysts were detected in 2 eyes (4.9%). The myopic CNV area showed a mild and non-significant reduction. Subretinal fibrosis was visible in 10 eyes (24.4%) at M6, and in 19 eyes (46.3%) at M12. Eyes without subretinal fibrosis had better mean final BCVA than eyes with subretinal fibrosis (0.19 ± 0.24 logMAR vs 0.38 ± 0.22 logMAR, p = 0.012). A significant difference in patients’ age was seen between eyes with and without subretinal fibrosis (51.6 ± 18.3 vs 62.8 ± 13.1 years, p = 0.033). Complete OCT parameters data in patients affected by immature and mature mCNV are reported respectively in Tables 3 and 4.

**Table 3:** Results of repeated variables over the follow-up study period in eyes with mature mCNV (n = 21).

Variable	Baseline	1 month	6 months	12 months	p value
BCVA (logMAR)	0.60 ± 0.34	0.46 ± 0.31	0.36 ± 0.24^a^	0.42 ± 0.26	**0.046**^A^
Ellipsoid zone disruption (n)	19 (90.5%)	13 (61.9%)	1 (4.8%)^b^	2 (9.5%)^b^	**< 0.001**^Q^
Subretinal hyperreflective exudation (n)	19 (90.5%)	13 (61.9%)	1 (4.8%)^b^	0 (0%)^b^	**< 0.001**^Q^
Intraretinal cysts (n)	9 (42.9%)	10 (47.6%)	4 (19.0%)	1 (4.8%)^c^	**0.001**^Q^
Subretinal fluid (n)	11 (52.4%)	2 (9.5%)^d^	0 (0%)^b^	2 (9.5%)^d^	**< 0.001**^Q^
CNV area (mm^2^)	1.01 ± 0.53	0.92 ± 0.50	0.83 ± 0.41	0.85 ± 0.41	0.618^A^
Subretinal fibrosis (n)	0 (0%)	0 (0%)	9 (42.9%)^e^	17 (81%)^f^	**< 0.001**^Q^

Significant values are in bold.

^A^ANOVA test; ^Q^Cochran’s Q test; ^a^0.046 (Tukey HSD vs baseline); ^b^< 0.001(McNemar vs baseline); ^c^0.009 (McNemar vs baseline); ^d^0.012 (McNemar vs baseline); ^e^0.027 (McNemar vs baseline); ^f^0.002 (McNemar vs baseline).

*BCVA* Best-Corrected Visual Acuity, *CNV* Choroidal neovascularization, *logMAR* logarithm of the minimal angle of resolution, *mm* millimeter.

**Table 4:** Results of repeated variables over the follow-up study period in eyes with immature mCNV (n = 20).

Variable	Baseline	1 month	6 months	12 months	p value
BCVA (logMAR)	0.35 ± 0.29	0.21 ± 0.20	0.16 ± 0.18^a^	0.14 ± 0.15^b^	**0.014**^A^
Ellipsoid zone disruption (n)	18 (90%)	7 (35%)^c^	2 (10%)^d^	2 (10%)^d^	** < 0.001**^Q^
Subretinal hyperreflective exudation (n)	20 (100%)	6 (30%)^d^	2 (10%)^d^	1 (5%)^d^	** < 0.001**^Q^
Intraretinal cysts (n)	2 (10%)	0 (0%)	0 (0%)	1 (5%)	0.300^Q^
Subretinal fluid (n)	6 (30%)	1 (5%)	1 (5%)	0 (0%)^e^	**0.012**^Q^
CNV area (mm^2^)	0.18 ± 0.19	0.14 ± 0.20	0.11 ± 0.12	0.11 ± 0.13	0.636^A^
Subretinal fibrosis (n)	0 (0%)	0 (0%)	1 (24.4%)	2 (46.3%)	0.194^Q^

Significant values are in bold.

^A^ANOVA test; ^Q^ Cochran’s Q test; ^a^0.035 (Tukey HSD vs baseline); ^b^0.017 (Tukey HSD vs baseline); ^c^0.001(McNemar vs baseline); ^d^0.001 (McNemar vs baseline); ^e^0.016 (McNemar vs baseline).

*BCVA* Best-Corrected Visual Acuity, *CNV* Choroidal neovascularization, *logMAR* logarithm of the minimal angle of resolution; *mm* millimeter.

Results of univariate regression analyses of factors influencing final BCVA and subretinal fibrosis are listed in Table 5.

**Table 5:** Results of univariate regression analysis for BCVA at 12 months and Subretinal Fibrosis occurrence at 12 months as dependent variables.

Variable	BCVA at 12 months		Subretinal fibrosis at 12 months	
	Beta	p	Beta	p
Gender	0.202	0.205	**0.318**	**0.043**
Age	0.149	0.351	**− 0.344**	**0.028**
CNV immaturity	**− 0.533**	** < 0.001**	**− 0.711**	** < 0.001**
CNV location	**− **0.099	0.536	**− **0.153	0.338
Axial length	0.018	0.911	0.114	0.477
Number of injections	**0.498**	**0.001**	**0.415**	**0.007**
Baseline BCVA	**0.863**	** < 0.001**	**0.377**	**0.015**
Baseline hemorrhages	0.611	0.284	0.252	0.525
Baseline EZ disruption	**0.337**	**0.031**	**− **0.024	0.881
Baseline intraretinal cysts	**0.303**	**0.054**	**− **0.011	0.947
Baseline subretinal fluid	0.190	0.234	**0.211**	**0.186**
Baseline SHE	**0.209**	**0.190**	**− 0.244**	**0.125**
FA pattern	0.186	0.243	**0.329**	**0.036**
CNV area	**0.425**	**0.006**	**0.625**	** < 0.001**
M1 BCVA	**0.872**	** < 0.001**	**0.428**	**0.005**
M1 EZ disruption	**0.608**	** < 0.001**	**0.365**	**0.019**
M1 intraretinal cysts	**0.400**	**0.010**	**0.269**	**0.088**
M1 subretinal fluid	**− **0.088	0.585	**0.302**	**0.055**
M1 SHE	**0.644**	** < 0.001**	**0.411**	**0.008**
M1 CNV area	0.451	0.003	**0.627**	** < 0.001**
M6 BCVA	**0.943**	** < 0.001**	**0.369**	**0.018**
M6 EZ disruption	0.159	0.321	**0.036**	0.823
M6 intraretinal cysts	**0.399**	**0.010**	**0.354**	**0.023**
M6 subretinal fluid	**− **0.082	0.612	**− **0.147	0.359
M6 SHE	**− **0.107	0.506	**− **0.073	0.649
M6 CNV area	**0.488**	**0.003**	**0.605**	** < 0.001**
M6 SF	**0.238**	**0.134**	0.611	–
M12 BCVA		–	**0.386**	**0.013**
M12 EZ disruption	0.014	0.929	**− **0.141	0.380
M12 intraretinal cysts	0.136	0.395	0.017	0.918
M12 subretinal fluid	0.067	0.676	**0.244**	**0.125**
M12 SHE	**− **0.082	0.612	**− **0.147	0.359
M12 CNV area	**0.490**	**0.001**	**0.623**	** < 0.001**
M12 SF	**0.386**	**0.013**		–

Significant values are in bold.

Variables with a p value < 0.2 were included in the multivariate regression analysis.

*BCVA* Best-Corrected Visual Acuity, *CNV* choroidal neovascularization, *SF* subretinal fibrosis, *FA* fluorescein angiography, *SHE* subretinal hyperreflective exudation, *EZ* ellipsoid zone, *M1* 1-month examination, *M6* 6-month examination, *M12* 12-month examination.

Multivariate regression analyses showed that baseline factors related to better BCVA at 12 months were better baseline BCVA (p < 0.001) and immature mCNV pattern (p = 0.005). Multivariate analyses of factors influencing final BCVA at every single follow-up visit are reported in Table 6.

**Table 6:** Results of multivariate regression analysis for BCVA at 12 months as dependent variables.

	Beta	p
**Baseline parameters**		
BCVA	0.773	< 0.001
Immature mCNV pattern	− 0.237	0.005
**1-month parameters**		
BCVA	0.826	< 0.001
**6-month parameters**		
BCVA	0.943	< 0.001
**12-month parameters**		
mCNV area at M12	0.490	0.001

*BCVA* Best-Corrected Visual Acuity, *mCNV* myopic choroidal neovascularization, *M1* 1-month examination, *M6* 6-month examination, *M12* 12-month examination.

Multivariate regression analyses showed that baseline factors related to subretinal fibrosis occurrence at 12 months were immature CNV pattern (p < 0.001) and younger age (p = 0.047). The multivariate regression analyses of follow-up visits parameters are listed in Table 7.

**Table 7:** Results of multivariate regression analysis for Subretinal Fibrosis occurrence at 12 months as dependent variables.

	Beta	p
**Baseline parameters**		
Immature mCNV pattern	− 0.672	< 0.001
Age	− 0.226	0.047
**1-month parameters**		
mCNV area at M1	0.535	< 0.001
SRF at M1	0.274	0.025
**6-month parameters**		
mCNV area at M6	0.605	< 0.001
**12-month parameters**		
mCNV area at M12	0.614	< 0.001

*BCVA* Best-Corrected Visual Acuity, *mCNV* myopic choroidal neovascularization, *M1* 1-month examination, *M6* 6-month examination, *M12* 12-month examination.

## Discussion

In this study we investigated the functional and morphological changes of mCNV in patients treated with anti-VEGF injections over a 12-month longitudinal follow-up period.

Randomized controlled trials on myopic CNV have shown positive functional outcomes with anti-VEGF drugs^11,12^. In our study, mean BCVA improved from 0.48 logMAR to 0.28 logMAR, and after 1 year of follow-up, 48.8% of the eyes (20/41) had a BCVA > 20/40, indicating the great efficacy of anti-VEGF treatment. Hayashi et al., in a study on the natural course of mCNV, found a mean BCVA of 0.83 logMAR after 1 year; after 5 years of follow-up, only 14% of patients had a BCVA > 20/40^13^, highlighting how poor the long-term functional outcomes are without anti-VEGF treatment.

Several SD-OCT morphological biomarkers related to myopic CNV activity, such as SHE, SRF and IRC, have been employed in clinical practice. A strong correlation between presence of SHE with fuzzy borders and fluorescein leakage has been demonstrated^14,15^. Conversely, signs of OCT fluid accumulation are less frequently detected, thus representing a less accurate sign of CNV activity^16^. Cohen et al. reported baseline hyperreflective exudation, SRF and IRC rates of 100%, 31% and 82,7% respectively, which had decreased to 10.3%, 6.9% and 6.9% respectively, six months after the treatment. Similar outcomes were detected by Bruyerre et al., who found rates of 100%, 25.8% and 41.9% at baseline, and lower rates at the 6-month follow-up visit.

We found a progressive reduction in the rate of eyes with CNV activity signs up to 12 months, suggesting that the treatment determines a long-term stabilization of the CNV. In clinical practice, strict monthly follow-up visits are needed to promptly re-treat the patients, based on the mCNV activity. Thus, it is essential to employ non-invasive imaging techniques, and apart from the OCT signs, several studies have proven OCTA’s great sensitivity and specificity in detecting CNV activity by analysing mCNV features^7,17,18^.

The pattern of the CNV, evaluated by OCTA, shows great clinical relevance. Bruyere et al. supposed three stages of CNV life-cycle, starting with an immature stage (small, disorganized, neovascular network), a mature stage (larger, structured mCNV) and the final fibrotic evolution. In our study, at final evaluation, one third of immature mCNV had totally disappeared. Overall, immature CNVs had a lower duration (16.1 days vs 23 days), greater final BCVA (0.14 ± 0.15 logMAR vs 0.40 ± 0.26 logMAR) and required less injections than mature CNVs. Multivariate regression analysis showed that the immature CNV pattern and baseline BCVA were predictive factors for healthy final BCVA, strongly supporting the importance of an early diagnosis to promptly start the treatment when BCVA is still preserved^19^.

An alternative interpretation in neovascular AMD hypothesized that two neovascular forms can be identified: arteriolar CNV (well defined pattern) and capillary CNV (small and ill-defined pattern) which are two distinct pathobiological entities with specific subtype of neovessel generally conserved over time^20^. However, both the speculations support the importance of OCTA as imaging tool to identify responder patients by assessing mCNV features.

Myopic CNV typically affects the middle-aged population with a long-life expectancy. Then, it is of great clinical relevance to identify morphologic predictive factors that may help in the long-term management of mCNV patients. The clinical relevance of mCNV size was noteworthy even before the advent of OCTA^21^, and small CNV size evaluated by fluorescein angiography was associated with desirable visual acuity outcomes^13^. In our study, we observed that the final CNV area, assessed by OCTA, was the factor most influencing the final BCVA. Subretinal fibrosis at 12 months showed a strong association with the final BCVA at univariate regression analysis (p = 0.013), however this association was lost at multivariate analysis where the 12-month CNV area was included. Xiao et al. detected subretinal fibrosis at 12 months in 40.7% of eyes, with higher incidence in subfoveal CNV^22^. In our analysis, the rate of subretinal fibrosis at 12 months was 46.34%, and 25% of our patients had subretinal fibrosis at 6 months. We found a close association of subretinal fibrosis with the CNV pattern, age, the presence of subretinal fluid at 1 month and the CNV area at 1, 6 and 12-moth examination. Other studies found that profuse leakage is associated with higher rates of exudation and subretinal fibrosis may indicate a more advanced stage of CNV maturation^23^. We found that the FA pattern was correlated to fibrosis in univariate regression analysis, but not in multivariate regression analysis when OCT and OCTA parameters were included, suggesting that the latter may be more capable of predicting fibrosis occurrence.

In this study, we observed that the immature CNV pattern and a small final CNV area are associated to better final BCVA. Mature CNVs, with a higher mCNV duration, require more anti-VEGF injections to achieve disease stabilization and are associated with a poorer final visual outcome. No association was observed at multivariate analysis between number of injections and subretinal fibrosis while a significant difference in the number of injections between mature and immature CNV was observed (2.5 vs 3.8 p = 0.002).

The identification of clinical and morphological factors predicting fibrosis at 12 months has a long-term prognostic implication because subjects with subretinal fibrosis have a higher risk of advanced myopic maculopathy and chorioretinal atrophy, which is the main cause of long-term visual decline^24^. Moreover, in our analysis, subretinal fibrosis was associated with a younger age, confirming previous findings by Xiao et al.

In this study we integrated FA, SD-OCT and OCTA biomarkers to investigate mCNV changes and the morpho-functional correlations during the treatment period with a 12-month longitudinal follow-up. This approach will help to better understand the pathogenesis of inflammatory mCNV and the mechanisms leading to subretinal fibrosis. OCTA shows an essential clinical role in facilitating the diagnosis and obtaining clinical information in a non-invasive fashion.

This study has several limitations, including the need for high-quality imaging to get information by OCTA examination, the monocentric setting and the limited number of eyes.

In conclusion, our study shows that patients with immature mCNVs have a better functional outcome and require a lower number of injections. OCTA is useful to predict subretinal fibrosis occurrence and it provides prognostic information in the management of mCNV which cannot be achieved by other imaging techniques.

## Materials and methods

Forty patients (41 eyes) affected by mCNV were recruited in this 12-month prospective study from July 2018 to January 2020 at the Eye Clinic of the University of Catania. The study adhered to the tenets of the Helsinki declaration and was approved by the Comitato Etico Catania 1 (Local Institutional Review Board). Informed consent was obtained from all subjects and/or their legal guardian(s).

Inclusion criteria were: axial length (AL) > 26 mm or refractive error (spherical equivalent) > − 6 Diopters (D) associated with clinical changes typical of PM, diagnosis of subfoveal or juxtafoveal/extrafoveal naïve mCNV, high-quality imaging. Exclusion criteria included any other retinal diseases, previous ocular surgery (except for cataract extraction performed more than 6 months earlier), any systemic condition which could interfere with the treatment and inferior-quality imaging.

All patients underwent a complete baseline ophthalmic examination including BCVA test, biomicroscopy of the anterior segment, fundus examination in mydriasis, OCT, OCTA and FA. All patients underwent one ranibizumab intravitreal injection at baseline; additional intravitreal injections were administered over the follow-up period on the basis of the activity signs detected by OCT, OCTA and FA, with a pro-re-nata (PRN) treatment protocol.

Patients were re-examined 1, 6 and 12 months after the first intravitreal injection; additional visits were performed according to clinical management needs. For each visit patients received all the examinations, except FA, which was performed whenever deemed necessary.

BCVA was tested on standard Early Treatment Diabetic Retinopathy Study (ETDRS) charts converted into logarithm scale of the minimal angle of resolution (logMAR). OCT and FA were carried out by means of Spectralis HRA + OCT with a confocal scanning laser ophthalmoscope (Heidelberg Engineering, Heidelberg, Germany). The OCT scan protocol used was a 15° × 15° 67-section macular cube with additional high resolution horizontal and vertical single lines centred on the CNV. AngioVue XR Avanti (Optovue Inc, Fremont, California, USA) was used to perform OCTA. The A-scan rate was 70,000 per second, with a bandwidth of 50 nm and a light source of 840 nm. The OCTA macula images (6 mm × 6 mm) obtained were centered on the foveola. Each volume contained 400 × 400 A-scans with 2 consecutive B-scans acquired in each fixed position. The split-spectrum amplitude-decorrelation angiography method was used to capture the dynamic motion of red blood cells. Using AngioAnalytics software, mCNV areas on the outer retina slab were selected manually. The values of the “CNV select area” were included in the analysis as mCNV size. The Central Foveal Thickness (CFT, micron, μ) was automatically provided by the machine.

Signs of mCNV activity on OCT including intraretinal cysts (IRC, defined as presence of intraretinal areas of low reflectivity), subretinal fluid (SRF, defined as areas of low reflectivity in the subretinal space), ellipsoid zone disruption (EZ disruption, defined as the loss of integrity of the EZ reflectivity) and subretinal hyperreflective exudation with fuzzy borders (SHE, characterized by hyperreflective material beneath the neurosensory retina) were assessed at baseline and at follow-up visits.

FA patterns were defined as profuse leakage (intense diffusion of the dye beyond the CNV border) or minimal leakage (slight leakage through CNV edges in late frames) according to the leakage detectable 5 min after the intravenous dye injection^23^.

**Figure 1:**
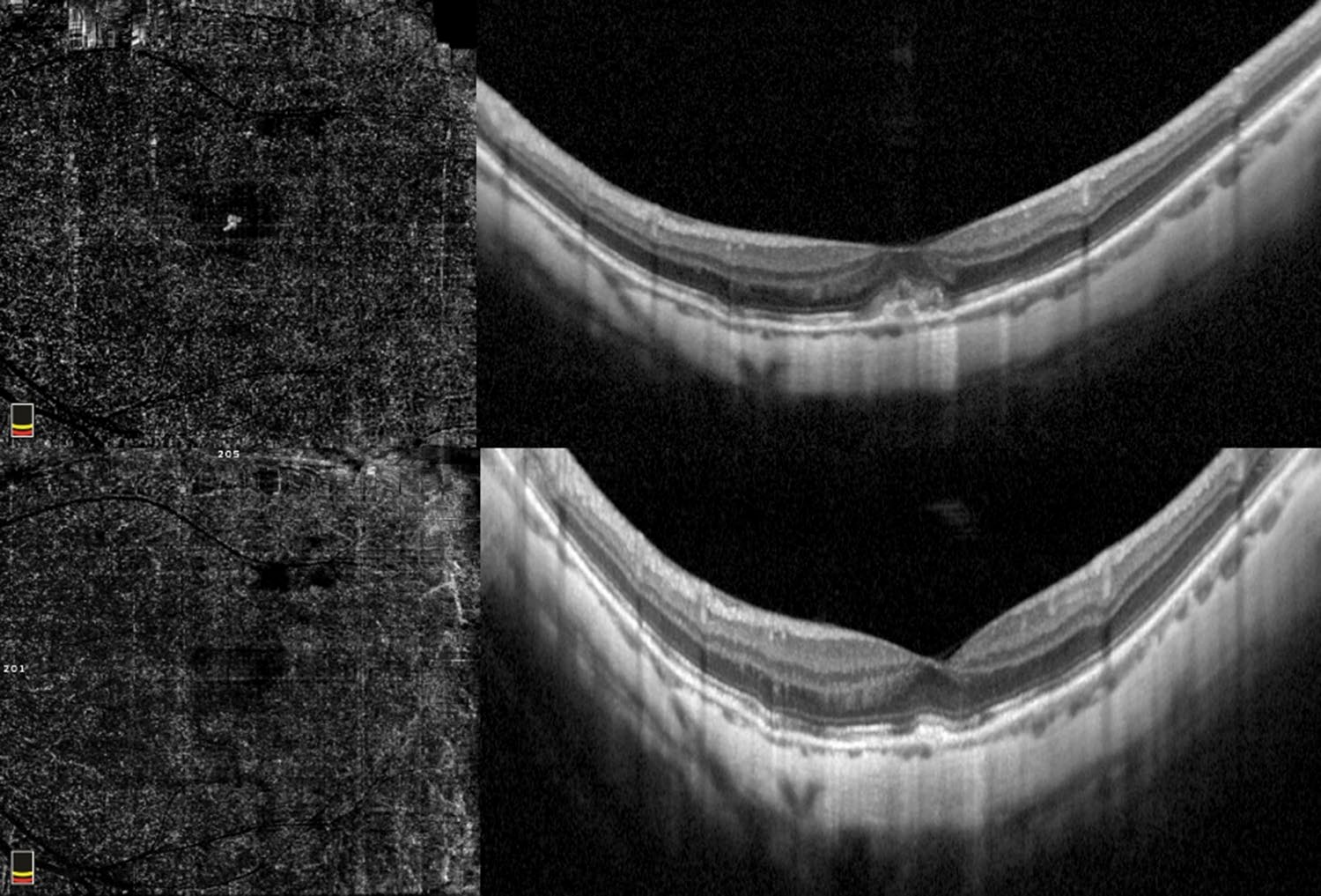
Immature myopic CNV. Baseline OCTA examination of a case of immature subfoveal myopic CNV (top left). The corresponding SD-OCT image shows the hyperreflective subretinal exudation with fuzzy borders, absence of intraretinal cysts and subretinal fluid (top right). OCTA at the final visit (12 months) after two intravitreal injections highlight the complete regression of the neovascular network (bottom left) and SD-OCT scan shows the disappearance of the subretinal material (bottom right).

**Figure 2:**
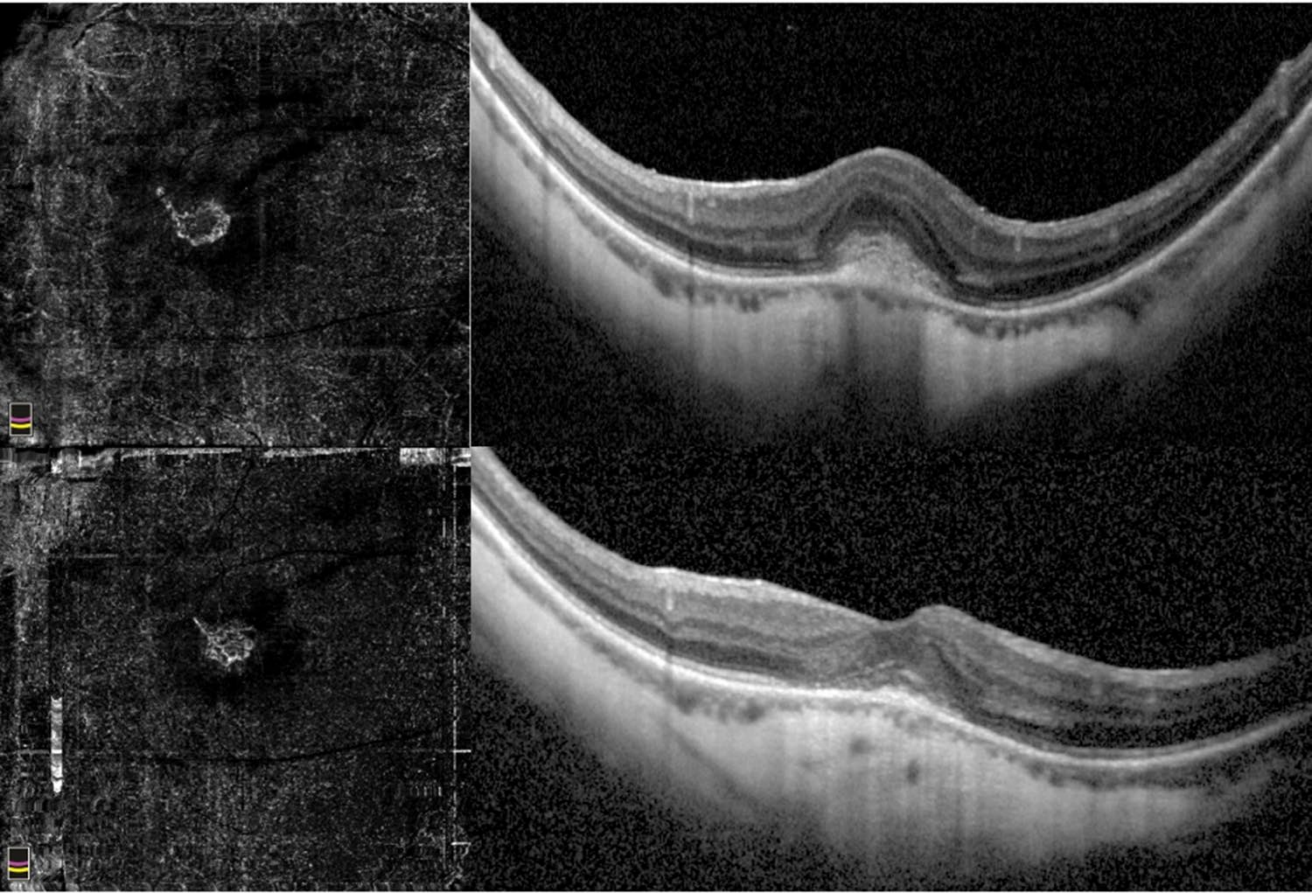
Mature myopic CNV. Baseline OCTA examination of a case of mature myopic CNV (top left) with wide subretinal hyperreflective exudation with fuzzy borders and absence of fluid accumulation signs assessed by SD-OCT (top right). The neovascular network at the final visit (12 months) after 5 intravitreal injections assessed by OCTA is still visible and shows a slight reduction of the size (bottom left). The SD-OCT exudation signs are regressed with absence of intraretinal cysts and subretinal fluid (bottom right).

Myopic CNV was categorized either as subfoveal or juxtafoveal/extrafoveal according to the location. Moreover, mCNV was categorized, using OCTA according to previously described classifications, either as immature (small-size, disorganized vascular loops without an evident interlacing vascular network), or mature (larger, highly structured, characterized by an interlacing pattern of the new vessel network with a large CNV select area) (Figs. 1 and 2, respectively)^25^. Subretinal fibrosis was defined as the fundoscopic detection of greyish or yellowish subretinal tissue underneath the retina in the foveal or parafoveal area, corresponding to an OCT image of a well-demarcated hyperreflective lesion situated between the retinal pigment epithelium and the neurosensory retina.

OCT, OCTA and FA parameters were evaluated by two independent examiners (N.C. and A.L.), whereas questionable cases were discussed with a third, trained examiner (T.A.).

The primary outcome measure was the identification of mCNV changes over the first year of anti-VEGF treatment. Secondary outcomes included the identification of factors influencing final BCVA and subretinal fibrosis occurrence.

The following parameters were included in the analysis: sex, age, presence of baseline macular haemorrhage, mCNV duration (based on referred symptoms between the onset and the first injection), mCNV location (subfoveal or juxtafoveal/extrafoveal), FA pattern (minimal/profuse), number of injections and Axial Length (AL, mm). Myopic CNV patterns (mature/immature) were assessed at baseline and at the final visit. The presence of SF was evaluated at follow-up visits. At every timepoint, BCVA, OCT activity signs (SHE, EZ disruption, SRF and IC) and CNV area (mm^2^) were evaluated.

Statistical analysis was performed by SPSS (version 22, IBM, New York, USA). Agreement between observers in mature/immature CNV was tested by Cohen’k. Comparison of continuous variable values between two groups was performed by unpaired t-test. Correlation was explored by Pearson’s *r*. ANOVA was used to compare values detected at different time-points for continuous variables. Post-hoc analysis was carried out by Tukey HSD test. For categorical variables, Cochran’s Q test was used; if significant, pairwise comparisons were made. Factors related to BCVA at 12 months or SF at 12 months (dependent variables), were tested in a univariate regression analysis. Variables with a p-value < 0.2 in univariate analysis, were included in multivariate regression analysis. P-values lower than 0.05 were considered as statistically significant.

## Data Availability

The datasets used and/or analysed during the current study are available from the corresponding author on request.
